# Mediation of the effects of living in extremely poor neighborhoods by health insurance: breast cancer care and survival in California, 1996 to 2011

**DOI:** 10.1186/1475-9276-12-6

**Published:** 2013-01-14

**Authors:** Kevin M Gorey, Isaac N Luginaah, Eric J Holowaty, Guangyong Zou, Caroline Hamm, Madhan K Balagurusamy

**Affiliations:** 1 School of Social Work, University of Windsor, 401 Sunset Avenue, Windsor, Ontario, N9B 3P4, Canada; 2 Department of Geography, University of Western Ontario, London, Ontario, Canada; 3 Dalla Lana School of Public Health, University of Toronto, Toronto, Ontario, Canada; 4 Department of Epidemiology and Biostatistics, University of Western Ontario and Scientist, Robarts Research Institute, London, Ontario, Canada; 5 Medical Oncologist, Windsor Regional Cancer Center, School of Medicine and Dentistry, Department of Medicine, Division of General Internal Medicine, University of Western Ontario, London, Ontario, Canada; 6 Statistician and Research Associate, School of Social Work, University of Windsor, Windsor, Ontario, Canada

**Keywords:** Health insurance, Poverty, Breast cancer care, Survival, Social capital, Health care reform, California, United States

## Abstract

**Background:**

We examined the mediating effect of health insurance on poverty-breast cancer care and survival relationships and the moderating effect of poverty on health insurance-breast cancer care and survival relationships in California.

**Methods:**

Registry data for 6,300 women with breast cancer diagnosed between 1996 and 2000 and followed until 2011 on stage at diagnosis, surgeries, adjuvant treatments and survival were analyzed. Socioeconomic data were obtained for residences from the 2000 census to categorize neighborhoods: high poverty (30% or more poor), middle poverty (5%-29% poor) and low poverty (less than 5% poor). Primary payers or health insurers were Medicaid, Medicare, private or uninsured.

**Results:**

Evidence of survival mediation was observed for women with node negative breast cancer. The apparent effect of poverty disappeared in the presence of Medicare or private health insurance. Women who were so insured were advantaged on 8-year survival compared to the uninsured or those insured by Medicaid (OR = 1.89). Evidence of payer moderation by poverty was also observed for women with node negative breast cancer. The survival advantaging effect of Medicare or private insurance was stronger in low poverty (OR = 1.81) than it was in middle poverty (OR = 1.57) or in high poverty neighborhoods (OR = 1.16). This same pattern of mediated and moderated effects was also observed for early stage at diagnosis, shorter waits for adjuvant radiation therapy and for the receipt of sentinel lymph node biopsies. These findings are consistent with the theory that more facilitative social and economic capital is available in low poverty neighborhoods, where women with breast cancer may be better able to absorb the indirect and direct, but uncovered, costs of care. As for treatments, main protective effects as well as moderator effects indicative of protection, particularly in high poverty neighborhoods were observed for women with private health insurance.

**Conclusions:**

America’s multi-tiered health insurance system mediates the quality of breast cancer care. The system is inequitable and unjust as it advantages the well insured and the well to do. Recent health care reforms ought to be enacted in ways that are consistent with their federal legislative intent, that high quality health care be truly available to all.

## Background

Studies of cancer care and survival in low-income areas of Canada and the United States have consistently observed Canadian advantages over the past generation [1-5]. More inclusive health insurance in Canada has been advanced as the most plausible explanation. These studies were ecological with respect to the measurement of socioeconomic status (SES). They used census tract data to define low-income neighborhoods, however, their lowest income areas typically only ranged from 10% to 20% poor. So they had limited power to study cancer care among “the truly disadvantaged” that live in America’s poorest neighborhoods where 30% to 40% or more of the households have incomes below the poverty line [6-8].

Recent analyses have suggested several reasons for the importance of investigating breast cancer care in such vulnerable places [2,5]. First, breast cancer is the most common cancer among American women and its prognosis is typically excellent with early diagnosis and treatment [9,10]. Second, a direct association between income and breast cancer survival has been consistently observed [2,11]. Third, as increasingly effective screens and treatments have proliferated evidence has mounted that the best care is more accessible to women of higher SES [3-5,12-17]. Fourth, being underinsured has also been associated with less than optimum care [18-24]. These US studies have also for the most part had insufficient samples and statistical power to study the quality of breast cancer care among those at greatest risk of not receiving it; the very poor and the inadequately insured.

### Combined effects of being underinsured and living in extremely poor neighborhoods

International studies have allowed us to develop the theory that SES-cancer care relationships are probably mediated by health insurance [2,25-27]. It is also probable that within the multi-payer US health care system the effects of various health insurers interact with other resources in ways not yet studied. However, thus far this field has primarily focused on advancing knowledge about main effects. More complex hypotheses about mediation and moderation effects have, for the most part, not been formally tested [17]. A recent study of ours may have been the first to test such effects in American cancer care [27]. We found evidence that having health insurance significantly mediated or buffered the poverty-survival relationship for women with colon cancer, but not for men. We also found that poverty moderated the health insurance-survival relationship, again only for women. The advantaging effects of all health insurers, public and private, were significantly larger for women in low-poverty neighborhoods. The present study will examine these relationships among women with breast cancer.

#### Health insurance in transaction with social capital

Our SES-health insurance-cancer care mediation theory aims to model care gaps in America’s most vulnerable communities. Its foundation is William Julius Wilson’s work in 1960s Chicago’s high poverty neighborhoods and Paul Jargowsky and Mary Jo Bane’s research in America’s extremely poor neighborhoods in the 1970s and 80s [6-8]. Together they described places of prevalent demographic vulnerability that were particularly distressed for their lack of social and economic capital. Medicaid and Medicare, the government’s, respective, mean-tested and entitlement, health insurance programs were specifically designed to mediate the effects of such impoverishment. But SES and public and private health insurance probably interact in important ways across America’s economic divide. A recent survey demonstrated how SES and health insurance can interact in the lives of people with cancer. It surveyed mostly insured people with colon cancer and found that more than a third of them suffered one or more significant financial hardships as a result of their treatment [28]. Moreover, more than one of every ten of them reported coverage denials by their insurers with consequent inabilities to adhere to prescribed treatment regimes. And low-income households were much more likely to be so impacted. It seems that the effectiveness of various health insurance programs may be significantly moderated by other resources. Those most likely to be disadvantaged are the poor. It is they who are the least likely to be able to bare the indirect (time lost from work, child care and travel) and direct, but uncovered costs (deductibles, co-insurances and co-payments) of contemporary cancer care in America [29].

#### Hypotheses

The objective of this study was to evaluate the distinct mediating and moderating effects of SES and health insurance on cancer care in extremely poor communities in the US. We hypothesized that the inverse poverty-breast cancer survival relationship would be significantly, if not completely, mediated or explained by the intermediate effect of having health insurance (private, Medicare or Medicaid). We also hypothesized a health insurance by poverty interaction, that is, that the direct insurance-breast cancer survival relationship would be significantly moderated by poverty (all types of health insurance would be less effective in high poverty neighborhoods) [30,31]. We then explored similar hypotheses across breast cancer care from diagnoses to adjuvant or palliative therapies.

## Methods

The sampling frame was the California cancer registry (CCR) which validly monitors the most populous US state [32-34]. California seems an appropriate place for this study as its population in high poverty neighborhoods nearly doubled between 1990 and 2000 while the nation’s population in high poverty neighborhoods decreased significantly [35,36]. Registry data was obtained for 6,300 women with invasive breast cancer diagnosed between 1996 and 2000 [37] and followed until 2011. Cases were randomly selected from three geographic place strata (2,100 cases each): megalopolises (greater San Diego, the San Francisco Bay area and greater Los Angeles), smaller metropolitan areas (Salinas, Modesto, Stockton, Bakersfield and Fresno) and rural places with population densities less than 400 per km^2^[38-40]. This study was powered to detect rate differences of 5% between 3 geographic place and 3 socioeconomic strata with 80% power at a 2-tailed significance level of 5% [41,42].

### Variables

#### Poverty and payers

We first linked breast cancer patients to the US (2000) census by their residential census tracts [38]. Next, to model our poverty measure after those that have been the most validated, we defined the following neighborhoods: high poverty (30% or more of households poor), middle poverty (5%-29% poor) and low poverty (less than 5% poor) [2,5-8,43-45]. Socioeconomic distributions of our sample’s poverty tertiles are displayed in Table 1. The prevalence of poverty in the typical high poverty neighborhood was 37%, just about mid-point between Wilson’s (30%) and Jargowsky and Bane’s (40%) criterion definitions of high poverty. The typical annual household income of $23,275 in such high poverty neighborhoods in 2000 seems a face valid definition of an extremely poor household. Most of this field’s heretofore low-income or “poor” samples would have fell into this study’s middle poverty group.

**Table 1:** **Prevalence of poor households in breast cancer** p**atients’ neighborhoods: California, 2000**

**Neighborhood poverty**	**Prevalence (%) of households**		
	**Living in poverty**		
	** Range**	** Median**	**Income,**^**a **^**$**
Low	0.00–4.99	3.36	74,000
Middle	5.00–29.76	11.40	44,200
High	30.09–100.00	36.84	23,275

Note. Neighborhood income derived from US Census data [38].

^a^ Census tract median annual household income.

Health insurance status, defined as the primary source of payment to the hospital or primary payer, was determined from medical records during the initial course of treatment. It was categorized as uninsured, Medicaid, Medicare (with or without any supplementation or any other non-means-tested governmental payer) or private insurer (any commercial managed care corporation or fee-for-service provider). The two key study variables of neighborhood poverty and primary payer are cross-tabulated in Table 2. Women with breast cancer living in high poverty California neighborhoods were one and a half times as likely to be uninsured, more than twelve times as likely to be primarily insured by Medicaid, but only two-thirds as likely to be covered by a private insurer as were their counterparts in low poverty neighborhoods.

**Table 2:** **Primary payers among women with breast cancer in low, middle and high poverty neighborhoods: age-adjusted prevalence** e**stimates and standardized prevalence ratios**

**Neighborhood poverty**				
**By primary payer**	**Sample**	**Prevalence**	**PR**	**(95% CI)**
Low Poverty				
Uninsured	183	.086	1.00	
Medicaid	25	.012	1.00	
Medicare	525	.263	1.00	
Private	1,367	.638	1.00	
Middle Poverty				
Uninsured	226	.108	**1.26**	(1.04, 1.52)
Medicaid	130	.062	**5.17**	(3.60, 7.42)
Medicare	577	.275	1.05	(0.94, 1.17)
Private	1,167	.555	**0.87**	(0.83, 0.91)
High Poverty				
Uninsured	276	.133	**1.55**	(1.30, 1.85)
Medicaid	310	.151	**12.58**	(9.28, 17.05)
Medicare	672	.307	**1.17**	(1.06, 1.29)
Private	842	.408	**0.64**	(0.60, 0.68)

Notes. *PR* = prevalence ratio, *CI* = confidence interval. A prevalence ratio of 1.00 was the between-place baseline. Middle and high poverty neighborhoods were compared to low poverty neighborhoods on payer prevalence estimates.

Bolded PRs were statistically significant at *p* < .05.

#### Breast cancer care

Variables from hospital and physicians’ office charts and clinic reports were coded by the CCR [46,47]. These were stage of disease at diagnosis (node negative, node positive or distally metastasized), receipt of initial surgery, type of surgery (lumpectomy or mastectomy), receipt of adjuvant radiation therapy, chemotherapy or hormone therapy, wait times from diagnosis to initial surgery and from surgery to adjuvant therapies, receipt of breast reconstruction surgery (post-mastectomy) and survival time from diagnosis to death or last follow-up at 10 years. All of these variables had less than 5% missing data. Tumor size (88.2%), histological grade (85.1%) and hormone receptor statuses (estrogen and progesterone receptors, 93.3%) were also coded for the vast majority of the sample. Various long wait criteria that may be associated with recurrences, metastases or shorter survival were explored [3,48-52].

Descriptive profiles of the breast cancer patients in our sample are displayed in Table 3. As planned, diverse geographic places, large urban to rural, and types of neighborhoods, high poverty to relatively affluent, were represented. Demographically, our age-diverse sample of younger working-age women (57.6%) and older women of retirement age (42.3%) seems consistent with expectations. More than half of them were primarily covered by a commercial, private insurer (53.6%). Given our oversampling of very poor neighborhoods, the relatively low representation of the uninsured (10.9%) may seem surprising. Note though that most initial breast cancer care takes place in hospitals where social workers work to connect uninsured patients to any additional resources, typically Medicaid or Medicare, for which they are qualified by virtue of being poor, older or disabled.

**Table 3:** Stratification, age, payer, staging, tumor and care characteristics of breast cancer patients diagnosed between 1996 and 2000 and followed until 2011

	**Sample**	**%**		**Sample**	**%**
Stratification characteristics					
Places			Poverty prevalence (%) in neighborhoods		
Large urban	2,100	33.3	< 5	2,100	33.3
Smaller urban	2,100	33.3	5–29	2,100	33.3
Rural	2,100	33.3	≥ 30	2,100	33.3
Age and primary payer characteristics					
Age at diagnosis, y			Primary payers		
25–44	904	14.3	Private insurers	3,376	53.6
45–54	1,386	22.0	Medicare	1,777	28.2
55–64	1,344	21.3	Medicaid	465	7.4
65–74	1,375	21.8	Uninsured	685	10.9
≥ 75	1,291	20.5			
Staging characteristics at diagnosis					
Summary stage			Size of tumor, mm		
Local-regional			< 10	975	17.5
Node negative	4,035	66.2	10–19	2,035	36.6
Node positive	1,774	29.1	20–49	2,035	36.6
Distally metastasized	287	4.7	≥ 50	512	9.2
Missing data	204	3.2	Missing data	743	11.8
Tumor characteristics					
Histological grade			Hormone receptor statuses		
I, well differentiated	1,134	21.1	Estrogen positive	3,720	63.3
II, moderately	2,211	41.2	Progesterone positive	3,189	54.3
III/IV, poorly	2,019	37.6	Either positive	3,852	65.5
Missing data	936	14.9	Missing data	422	6.7
Cancer care characteristics					
Surgery received			Wait time from diagnosis to surgery, d		
Lumpectomy	3,025	48.4	≤ 7	2,699	45.8
Mastectomy	2,876	46.0	8–30	2,011	34.1
No surgery	348	5.6	≥ 31	1,187	20.1
Missing data	51	0.8	Missing data	4	0.1
Received RT	2,843	45.4	Wait time after surgery for RT, d		
Missing data	44	0.7	≤ 60^a^	1190	42.9
			61–90	451	16.3
			91–180	687	24.8
			≥ 181	446	16.1
			Missing data	69	2.4
Received chemotherapy	2,299	37.6	Wait time after surgery for chemotherapy, d		
Missing data	185	2.9	≤ 30^b^	891	40.7
			31–60	839	38.3
			61–90	273	12.4
			≥ 91	188	8.6
			Missing data	108	4.7
Received HT	2,115	34.7	Wait time after surgery for HT, d		
Missing data	205	3.3	≤ 30^c^	701	34.7
			31–90	667	33.0
			91–180	408	20.2
			≥ 181	247	12.2
			Missing data	92	4.3

Note. *RT* = radiation therapy, *HT* = hormone therapy.

^a^ A few (41) of these patients received RT prior to or without surgery.

^b^ One third (32.4%) of these patients received chemotherapy prior to or without surgery.

^c^ One fifth (18.5%) of these patients received HT prior to or without surgery.

### Statistical analyses

We used logistic regression models to test hypotheses about the mediating and moderating effects of poverty and payers in predicting binary (survived or not) all-cause breast cancer survival [53-55]. Odds ratios (ORs) and 95% confidence intervals (CIs) were estimated. Survival outcomes that were best predicted by main poverty and payer effects and their interactions were analyzed and reported: 8-year for node negative, 5-year for node positive and 3-year for distally metastasized disease (Hosmer-Lemeshow test [55,56]). ORs estimate the relative predictive weights of main and interacting effects. However, under the circumstances of this study, where both social exposures and key outcomes are common, ORs probably overestimate rate ratios (RRs) [57]. So we provided accompanying practical assessments more germane perhaps to clinical and policy significance. Rates were directly adjusted for age and other relevant covariates that significantly and substantially confounded poverty- or payer-breast cancer care relationships, using this study’s sample as the standard and reported as rates per 100 cases or percentages. Then we used standardized RRs for between-group comparisons, with 95% CIs derived from the Mantel-Haenszel χ^2^ test [58,59]. Statistical model tests (ORs with 95% CIs) are presented in Tables 4 ,5, 6, 7 and 8 with accompanying “real world” practical significance indices (RRs with 95% CIs) reported as interpretive adjuncts in the text. Because some of our subsample-based hypotheses could be deemed exploratory we also reported as approaching significance any finding that met the more liberal statistical criterion of *p* < .10.

**Table 4:** Logistic regression main effects and interactions of neighborhood poverty and primary payer by breast cancer stage at diagnosis on survival

	**Local-Regional**						**Distally Metastasized**		
	**Node negative disease**			**Node positive disease**					
	**8 year survival**			**5 year survival**			**3 year survival**		
**Predictor Variables**	**Sample**	**OR**	**(95% CI)**	**Sample**	**OR**	**(95% CI)**	**Sample**	**OR**	**(95% CI)**
	Single predictor models								
Neighborhood poverty									
< 5% poor	1,435	1.00		561	1.00		55	1.00	
5-29% poor	1,353	**0.73**	(0.61, 0.88)	574	**0.70**	(0.53, 0.93)	97	0.76	(0.37, 1.59)
≥ 30% poor	1,247	**0.54**	(0.45, 0.65)	639	**0.41**	(0.31, 0.53)	135	0.63	(0.31, 1.29)
Primary payer									
Uninsured or Medicaid	572	1.00		382	1.00		84	1.00	
Medicare or private	3,463	**1.57**	(1.27, 1.96)	1,392	**1.86**	(1.44, 2.41)	203	**2.51**	(1.28, 4.92)
	Full models								
Neighborhood poverty									
< 5% poor	1,435	1.00		561	1.00		55	1.00	
5-29% poor	1,353	0.92	(0.67, 1.26)	574	**0.72**	(0.55, 0.96)	97	0.79	(0.38, 1.68)
≥ 30% poor	1,247	0.84	(0.50, 1.40)	639	**0.44**	(0.34, 0.58)	135	0.76	(0.36, 1.59)
Primary payer									
Uninsured or Medicaid	572	1.00		382	1.00		84	1.00	
Medicare or private	3,463	**1.89**	(1.26, 2.84)	1,392	**1.59**	(1.22, 2.07)	203	**2.40**	(1.21, 4.79)
Poverty by payer	4,035	0.79^*^	(0.60, 1.04)	1,774	1.20^a^	(0.85, 1.69)	287	1.17^a^	(0.39, 3.54)
	Poverty by payer interaction among women with node negative disease on 8 year survival								
	> 30% poor			5-29% poor			< 5% poor		
	Sample	OR	(95% CI)	Sample	OR	(95% CI)	Sample	OR	(95% CI)
Primary payer									
Uninsured or Medicaid	277	1.00		186	1.00		109	1.00	
Medicare or private	970	1.16	(0.82, 1.62)	1,167	**1.57**	(1.09, 2.27)	1,326	**1.81**	(1.11, 2.95)

Notes. *OR* = odds ratio, *CI* = confidence interval. All effects were age-adjusted across these categories: 25–44, 45–54, 55–64, 65–74 and 75 or older. After age, poverty and payer were accounted for, place (large urban, smaller urban or rural) and race/ethnicity (person of color [32.1%] or non-Hispanic white) did not enter any of the full models. Bolded ORs were statistically significant at *p* < .05.

^a^ Null interaction was removed from the full model. ^*^p < .10.

**Table 5:** Logistic regression main effects and interactions of neighborhood poverty and primary payer on breast cancer stage at diagnosis

	**Node negative disease**			**Tumor < 20 mm**		
**Predictor Variables**	**Sample**	**OR**	**(95% CI)**	**Sample**	**OR**	**(95% CI)**
Single predictor models						
Neighborhood poverty						
< 5% poor	1,833	1.00		1,704	1.00	
5-29% poor	1,760	0.94	(0.81, 1.08)	1,639	0.88^*^	(0.75, 1.02)
≥ 30% poor	1,723	**0.75**	(0.65, 0.88)	1,575	**0.61**	(0.52, 0.71)
Primary payer						
Uninsured or Medicaid	888	1.00		796	1.00	
Medicare or private	4,428	**1.42**	(1.21, 1.65)	4,122	**1.37**	(1.16, 1.61)
Full models						
Neighborhood poverty						
< 5% poor	1,833	1.00		1,704	1.00	
5-29% poor	1,760	1.27	(0.88, 1.84)	1,639	0.89	(0.76, 1.04)
≥ 30% poor	1,723	1.06	(0.74, 1.52)	1,575	**0.63**	(0.54, 0.73)
Primary payer						
Uninsured or Medicaid	888	1.00		796	1.00	
Medicare or private	4,428	**1.73**	(1.23, 2.44)	4,122	**1.23**	(1.04, 1.45)
Poverty by payer	5,316	0.72^*^	(0.49, 1.06)	4,918	1.16^a^	(0.93, 1.45)
Poverty by payer interaction on node negative disease at diagnosis						
	> 5% poor			< 5% poor		
Predictor Variables	Sample	OR	(95% CI)	Sample	OR	(95% CI)
Primary payer						
Uninsured or Medicaid	730	1.00		158	1.00	
Medicare or private	2,753	**1.28**	(1.07, 1.53)	1,675	**1.76**	(1.25, 2.48)

Notes. *OR* = odds ratio, *CI* = confidence interval. All effects were age and grade-adjusted across these categories: 25–44, 45–54, 55–64, 65–74 and 75 or older; and well, moderately or poorly differentiated. Bolded ORs were statistically significant at *p* < .05.

^a^ Null interaction was removed from the full model. ^*^p < .10.

**Table 6:** Logistic regression main effects and interactions of neighborhood poverty and primary payer on wait times from diagnosis to surgery and from surgery to radiation therapy among women with local-regional breast cancer

				**[No chemotherapy]**			**[Had chemotherapy]**		
	**Waited > 60 days**			**Waited > 90 days**			**Waited > 210 days**		
	**diagnosis to surgery**			**surgery to radiation therapy**			**surgery to radiation therapy**		
**Predictor variables**	**Sample**	**OR**	**(95% CI)**	**Sample**	**OR**	**(95% CI)**	**Sample**	**OR**	**(95% CI)**
	Single predictor models								
Neighborhood poverty									
< 5% poor	1,975	1.00		640	1.00		403	1.00	
5-29% poor	1,901	1.21	(0.92, 1.59)	507	1.06	(0.71, 1.57)	382	0.84	(0.59, 1.18)
≥ 30% poor	1,841	**1.88**	(1.46, 2.43)	382	**1.50**	(1.01, 2.25)	361	0.89	(0.63, 1.26)
Primary payer									
Uninsured or Medicaid	934	1.00		224	1.00		252	1.00	
Medicare or private	4,783	**0.61**	(0.48, 0.77)	1,305	**0.52**	(0.35, 0.77)	894	0.87	(0.62, 1.23)
	Full models								
Neighborhood poverty									
< 5% poor	1,975	1.00		640	1.00		403	1.00	
5-29% poor	1,901	1.95^*^	(0.94, 4.03)	507	1.02	(0.69, 1.52)	382	**1.92**	(1.02, 3.61)
≥ 30% poor	1,841	**2.86**	(1.40, 5.82)	382	1.37	(0.91, 2.06)	362	1.54	(0.83, 2.84)
Primary payer									
Uninsured or Medicaid	934	1.00		224	1.00		252	1.00	
Medicare or private	4,783	1.13	(0.56, 2.29)	1,305	**0.55**	(0.37, 0.82)	894	**0.65**	(0.42, 1.00)
Poverty by payer	5,717	0.52^*^	(0.25, 1.11)	1,529	1.03^a^	(0.64, 1.67)	1,146	1.96^*^	(0.97, 3.97)
	Poverty by payer interaction on surgical wait times of more than 2 months								
		> 5% poor				< 5% poor			
		Sample	OR	(95% CI)		Sample	OR	(95% CI)	
Primary payer									
Uninsured or Medicaid		765	1.00			169	1.00		
Medicare or private		2,977	**0.58**	(0.44, 0.75)		1,806	1.18	(0.58, 2.39)	
	Poverty by payer interaction among women who received chemotherapy on post-surgical wait times of more than 7 months for RT								
		> 30% poor				< 30% poor			
		Sample	OR	(95% CI)		Sample	OR	(95% CI)	
Primary payer									
Uninsured or Medicaid		121	1.00			131	1.00		
Medicare or private		240	0.96	(0.59, 1.57)		654	0.70^*^	(0.47, 1.03)	

Notes. *OR* = odds ratio, *CI* = confidence interval. All effects were age and stage-adjusted across these categories: 25–44, 45–54, 55–64, 65–74 and 75 or older; and node positive or node negative breast cancer. Bolded ORs were statistically significant at *p* < .05.

^a^ Null interaction was removed from the full model. ^*^p < 10.

**Table 7:** Logistic regression main effects and interactions of neighborhood poverty and primary payer on receipt of initial and adjuvant therapies among women with node negative breast cancer

	**Radiation therapy**^**a**^			**Chemotherapy**			**Hormone therapy**^**b**^		
**Predictor variables**	**Sample**	**OR**	**(95% CI)**	**Sample**	**OR**	**(95% CI)**	**Sample**	**OR**	**(95% CI)**
	Single predictor models								
Neighborhood poverty									
< 5% poor	877	1.00		1,327	1.00		932	1.00	
5-29% poor	762	0.80^*^	(0.63, 1.01)	1,232	1.18	(0.94, 1.46)	756	**0.77**	(0.63, 0.93)
≥ 30% poor	611	**0.63**	(0.49, 0.81)	1,113	0.96	(0.76, 1.22)	611	**0.70**	(0.56, 0.86)
Primary payer									
All others	980	1.00		1,627	1.00		1,002	1.00	
Private	1,270	**1.40**	(1.12, 1.75)	2,045	1.00	(0.81, 1.24)	1,297	**1.38**	(1.14, 1.66)
	Full models								
Neighborhood poverty									
< 5% poor	877	1.00		1,327	1.00		932	1.00	
5-29% poor	762	**0.62**	(0.44, 0.87)	1,232	1.16	(0.93, 1.44)	756	**0.78**	(0.64, 0.95)
≥ 30% poor	611	**0.51**	(0.37, 0.72)	1,113	0.77	(0.54, 1.10)	611	**0.73**	(0.59, 0.90)
Primary payer									
All others	980	1.00		1,627	1.00		1,002	1.00	
Private	1,270	0.97	(0.68, 1.40)	2,045	0.87	(0.66, 1.13)	1,297	**1.32**	(1.09, 1.60)
Poverty by payer	2,250	**1.61**	(1.05, 2.46)	3,672	1.43^*^	(0.93, 2.19)	2,299	0.98^c^	(0.79, 1.21)
	Poverty by payer interaction on receipt of adjuvant radiation therapy								
		> 5% poor			< 5% poor				
		Sample	OR	(95% CI)	Sample	OR	(95% CI)		
Primary payer									
All others		651	1.00		329	1.00			
Private		722	**1.56**	(1.19, 2.03)	548	1.06	(0.70, 1.61)		
	Poverty by payer interaction on receipt of chemotherapy								
		> 30% poor			< 30% poor				
		Sample	OR	(95% CI)	Sample	OR	(95% CI)		
Primary payer									
All others		639	1.00		988	1.00			
Private		474	1.39^*^	(0.94, 2.06)	1,571	0.86	(0.65, 1.13)		

Notes. *OR* = odds ratio, *CI* = confidence interval. All effects were age and tumor size-adjusted across these categories: 25–44, 45–54, 55–64, 65–74 and 75 or older; and less than 10, 10–19 and 20–50 mm or larger. Bolded ORs were statistically significant at *p* < .05.

^a^ Among women who received breast conserving surgery. ^b^ Among women with hormone receptor positive tumors.

^c^ Null interaction was removed from the full model. ^*^p < .10.

**Table 8:** Logistic regression main effects and interactions of neighborhood poverty and primary payer on receipt of initial and adjuvant therapies among women with non-distally metastasized, node positive breast cancer

	**Radiation therapy**			**Chemotherapy**^**a**^			**Hormone therapy**^**b**^		
**Predictor variables**	**Sample**	**OR**	**(95% CI)**	**Sample**	**OR**	**(95% CI)**	**Sample**	**OR**	**(95% CI)**
	Single predictor models								
Neighborhood poverty									
< 5% poor	497	1.00		364	1.00		364	1.00	
5-29% poor	525	0.82	(0.64, 1.05)	321	1.01	(0.73, 1.40)	333	1.19	(0.88, 1.62)
≥ 30% poor	568	**0.75**	(0.59, 0.96)	393	0.78	(0.56, 1.07)	335	0.90	(0.66, 1.23)
Primary payer									
Uninsured	174	1.00		107	1.00		103	1.00	
Any insurance	1,416	0.94	(0.76, 1.16)	931	1.12	(0.72, 1.75)	929	0.98	(0.64, 1.50)
	Full models								
Neighborhood poverty									
< 5% poor	497	1.00		324	1.00		364	1.00	
5-29% poor	525	0.82	(0.63, 1.05)	321	1.07	(0.77, 1.49)	333	1.32^*^	(0.96, 1.82)
≥ 30% poor	568	**0.73**	(0.57, 0.93)	393	0.86	(0.62, 1.21)	335	0.90	(0.66, 1.22)
Primary payer									
Uninsured	174	1.00		107	1.00		103	1.00	
Any insurance	1,416	0.87	(0.70, 1.09)	931	0.64	(0.32, 1.29)	929	0.68	(0.41, 1.15)
Poverty by payer	1,590	0.73^c^	(0.48, 1.12)	1,038	0.59^*^	(0.34, 1.01)	1,032	**0.33**	(0.13, 0.82)
	Poverty by payer interaction on receipt of chemotherapy								
		> 5% poor				< 5% poor			
		Sample	OR	(95% CI)		Sample	OR	(95% CI)	
Primary payer									
Uninsured		75	1.00			32	1.00		
Any insurance		639	1.63^*^	(0.91, 2.90)		292	0.55	(0.25, 1.17)	
	Poverty by payer interaction on receipt of hormone therapy								
	> 30% poor			5-29% poor			< 5% poor		
	Sample	OR	(95% CI)	Sample	OR	(95% CI)	Sample	OR	(95% CI)
Primary payer									
Uninsured	36	1.00		32	1.00		35	1.00	
Any insurance	299	0.72	(0.34, 1.52)	301	**2.16**	(1.00, 4.69)	329	0.59	(0.28, 1.26)

Notes. *OR* = odds ratio, *CI* = confidence interval. Effects on radiation and hormone therapies were age and tumor size-adjusted and effects on chemotherapy were adjusted for age and the receipt of radiation therapy across these categories: 25–44, 45–54, 55–64, 65–74 and 75 or older; less than 10, 10–19 and 20–50 mm or larger; and received radiation therapy or not. Bolded ORs were statistically significant at *p* < .05.

^a^ Among women who received mastectomies. ^b^ Among women with hormone receptor positive tumors.

^c^ Null interaction was removed from the full model. ^*^p < .10.

## Results

### Interacting effects of poverty and health insurance on breast cancer survival

Regression models for node negative, node positive and metastasized breast cancer survival are displayed in Table 4. Substantial support for both mediation and moderation hypotheses was observed for node negative disease. The top left column shows significant main effects of poverty (OR = 0.54) and payer (OR = 1.57) when these factors entered regression models alone. Moving down to the full model, consistent with mediation, in the presence of payer the effect of poverty disappeared and the effect of adequate payers was strengthened (OR = 1.89). The 8-year survival rate among those insured privately or by Medicare (75.5%) was nine percent greater than that of the uninsured or those covered by Medicaid (69.0%, RR = 1.09, 95% CI 1.03, 1.15).

The hypothesis that the health insurance-survival relationship would be moderated by poverty was also supported for women with node negative disease. The statistical interaction’s practical effect moderation is depicted in the bottom of Table 4. Having adequate health insurance seemed much more effective in low poverty (OR = 1.81, 95% CI 1.11, 2.95) than in high poverty neighborhoods, where Medicare or private insurance did not seem any more effective than having Medicaid or being uninsured (OR = 1.16, 95% CI 0.82, 1.62). Consistent with social capital theory, the 8-year survival rate among women with node negative breast cancer who lived in low poverty neighborhoods and were primarily covered by Medicare or private insurers (79.9%) was 13% greater than the survival rate among, otherwise similar, women primarily covered by Medicaid or who were uninsured (70.8%, RR = 1.13, 95% CI 1.02, 1.25). A somewhat smaller payer gradient was observed in middle poverty neighborhoods (75.5% vs 68.4%, RR = 1.10, 95% CI 1.01, 1.20) and no such gradient was observed in high poverty neighborhoods.

As for women with node positive and metastasized breast cancer, only significant main effects were observed. The practical 5-year survival effect of having Medicare or private coverage, versus Medicaid or none, for women with node positive disease was similar to that observed for women with node negative disease. Their respective survival rates were 73.6% and 66.2% (RR = 1.11, 95% CI 1.03, 1.19). And having such coverage seems to have made a very large difference in the lives of women diagnosed with metastasized disease. Those who were adequately covered were much more likely to have survived for 3 years (31.8%) than were the inadequately covered (18.2%, RR = 1.75, 95% CI 1.11, 2.75).

### Interacting effects of poverty and health insurance on breast cancer diagnoses and treatments

#### Stage at diagnosis

Mediation and moderation hypothesis support was also found for early diagnosis of node negative disease. In the full regression model, the apparent effect of living in high poverty neighborhoods disappeared, whereas the effect of payer (OR = 1.73) seemed strengthened and a poverty by payer interaction that approached statistical significance was detected (left column of Table 5). Evidence of this moderation of the effect of payer by poverty is depicted in the bottom of the table: low poverty (OR = 1.76) and middle to high poverty neighborhoods (OR = 1.28). In low poverty neighborhoods, women with Medicare or private health insurance were nearly 20% more likely (69.1%) to have been diagnosed with node negative disease than were the uninsured or those covered by Medicaid (58.3%, RR = 1.19, 95% CI 1.05, 1.34). Whereas the effectiveness of such health insurers was much smaller in middle to high poverty neighborhoods, where their respective early diagnosis rates were 65.3% and 61.5% (RR = 1.06, 95% CI 1.00, 1.13). As for tumor size at diagnosis, only significant main effects of high poverty (OR = 0.63) and payer (OR = 1.23) were observed, and the practical size of the payer-tumor size relationship was similar to that of the payer-early stage relationship.

#### Waits for care

Wait times for initial surgery and adjuvant radiation therapy of local-regional breast cancers are displayed in Table 6. Two findings are consistent with mediation and moderation hypotheses. First, payer clearly mediated the poverty-wait time relationship among women who received radiation therapy without chemotherapy as the apparent effect of poverty disappeared in the presence of the highly significant effect of payer (OR = 0.55, middle column of Table 6). In fact, the rate of waiting for more than three months after surgery for radiation therapy among those covered by Medicare or private insurers (13.5%) was less than half that of the uninsured or Medicaid covered (28.6%, RR = 0.47, 95% CI 0.36, 0.61). Second, poverty seemed to moderate the payer-wait time relationship among women who received adjuvant radiation therapy with chemotherapy as there was an effect of payer that approached statistical significance in middle and low poverty neighborhoods (OR = 0.70, *p* < .10), but not in high poverty neighborhoods (bottom of Table 6). The rate of waiting for more than seven months after surgery for radiation therapy among those covered by Medicare or private insurers (16.2%) was approximately 25% lower than that of the uninsured or Medicaid covered (21.8%, RR = 0.74, 95% CI 0.52, 1.06, *p* < .10).

Surgical wait times fit a different pattern. First, there was a very large effect of poverty (OR = 2.86). Women who lived in high poverty neighborhoods (12.7%) were two and a half times as likely to have waited two months or more for surgery than were women who lived in low poverty neighborhoods (5.0%, RR = 2.54, 95% CI 2.05, 3.15). Second, in the context of such a large effect of poverty, it seems that having adequate health insurance confers a protective effect in high and middle poverty (OR = 0.58), but not in low poverty neighborhoods. In higher poverty neighborhoods, adequately insured women were about a third less likely than were uninsured or Medicaid insured women to have waited two months or more. Such respective long wait rates were 6.9% and 10.5% (RR = 0.66, 95% CI 0.52, 0.96). Such long surgical wait rates were identical (5.5%) for both payer groups in low poverty neighborhoods.

#### Receipt of adjuvant therapies

The effects of poverty and payer on the receipt of adjuvant therapies for node negative and node positive breast cancers are, respectively, displayed in Tables 7 and 8. It should be noted that nearly all of them received initial surgery (98.5%) and such receipt was not associated with poverty or any payers. For women with node negative disease having private health insurance seemed to make the biggest difference and for women with node positive disease having any form of health insurance seemed to confer substantial access advantages over the uninsured. For women with node negative disease, in addition to having large main disadvantageous effects on access to radiation and hormone therapies, poverty seemed to moderate the effect that private insurance had in providing access to both adjuvant radiation and chemotherapies. Their poverty by payer interactions were, respectively, significant or nearly significant. The interactions depicted in the bottom of the table indicated protective effects conferred by having private health insurance specific to higher poverty neighborhoods. In high to middle poverty neighborhoods more than three-quarters of the women who had received breast conserving surgery and were privately insured received adjuvant radiation therapy (77.3%), whereas, only about two-thirds of otherwise similar women who were covered by Medicare, Medicaid or were uninsured did (67.6%, RR = 1.15, 95% 1.07, 1.23). No such effect was observed in low poverty neighborhoods. In high poverty neighborhoods there was an even larger effect of being privately insured on chemotherapy receipt. Respectively, 24.6% and 19.6% of privately insured and other insured or uninsured women received chemotherapy (RR = 1.26, 95% 1.00, 1.58). There was no significant effect of being privately insured in lower poverty neighborhoods. As for hormone therapy, only significant main effects of poverty and private insurance were observed. Again, having private insurance or not made a big difference in access to hormone therapy (50.7% versus 41.7%, RR = 1.22, 95% CI 1.12, 1.33).

For women with node positive disease having any form of health insurance seemed to confer substantial advantages over the uninsured. Poverty again moderated the effects of these payers on access to chemotherapy and hormone therapy (middle and right columns of Table 8). Large care gaps were observed among the uninsured, particularly among the uninsured that lived in the poorest neighborhoods. Such residents of high poverty neighborhoods who had any health insurance were much more likely to have received chemotherapy (85.5% vs 65.4%, RR = 1.31, 95% CI 1.16, 1.48). And such insured residents of middle poverty neighborhoods seemed more likely to have received hormone therapy (53.3% versus 36.6%, RR = 1.46, 95% CI 0.94, 2.27, *p* < .10). Not surprisingly, as it is not typically the first adjuvant treatment choice for node positive breast cancer, no significant payer effect was observed for radiation therapy.

#### Specialized care among women with non-metastasized disease

Next, we examined surgical procedures that came into practice (sentinel lymph node biopsy [SLNB]) or continued to proliferate during the study period (breast conserving surgery [BCS]) or that are not yet prevalently provided (breast reconstruction surgery [BRS]. There were similar patterns of Medicaid main effects and poverty by private insurance interactions for BCS and BRS. Those with Medicaid were less likely to receive BCS (37.1% vs 50.4%, RR = 0.74, 95% CI 0.65, 0.85) and much less likely to receive BRS (3.2% vs 13.7%, RR = 0.23, 95% CI 0.12, 0.46) than other women. And private insurance facilitated access to both BCS (50.2% vs 41.6%, RR = 1.21, 95% CI 1.07, 1.36) and BRS (12.9% vs 5.9%, RR = 2.19, 95% CI 1.55, 3.09) in high and middle poverty neighborhoods, but not in low poverty ones. Medicare or private health insurance completely mediated the disadvantaging effect of poverty on receipt of SLNB. Moreover, such coverage seemed to greatly facilitate access in low poverty neighborhoods (14.8% vs 6.6%, RR = 2.24, 95% CI 1.19, 4.20) more modestly facilitate access in middle poverty neighborhoods (10.8% vs 6.2%, RR = 1.74, 95% CI 1.04, 2.91), but seemed to have no effect in high poverty neighborhoods were very few women (6.3%) received such an innovative procedure.

#### Explorations of palliative care among women with metastasized disease

There seemed to be main effects of health insurance, but not poverty on the receipt of surgery and chemotherapy. Those with any insurance were about 50% more likely than the uninsured to have received surgery (46.7% vs 31.7%, RR = 1.47, 95% CI 0.94, 2.29, *p* < .10) and those with Medicare or private coverage were 25% more likely to have received chemotherapy than the uninsured or Medicaid covered (58.8% vs 47.2%, RR = 1.25, 95% CI 0.98, 1.60, *p* < .10). A protective effect of private insurance on the receipt of radiation therapy was only observed in middle poverty neighborhoods. More than half of such privately insured women received radiation therapy (52.6%), whereas only about a third of those covered by other than commercial insurers or who were uninsured did (35.3%, RR = 1.49, 95% CI 0.95, 2.35, *p* < .10). No significant main or interacting effects of health insurance were observed on hormone therapy. There was, however, quite a strong effect of poverty. Women in high poverty neighborhoods were only about half as likely to receive hormone therapy as were their counterparts in low poverty neighborhoods (36.4% vs 69.2%, RR = 0.53, 95% CI 0.33, 0.84).

## Discussion

We found strong support for our hypothesis that health insurance mediates the poverty-breast cancer survival relationship. Evidence of survival mediation was observed for women with the most common and treatable type of breast cancer, node negative. The effect of poverty disappeared in the presence of Medicare or private insurance. Women who were so insured were advantaged on survival compared to the uninsured or those insured by Medicaid. Evidence of insurance effect moderation by poverty was also observed for women with node negative disease. The survival advantaging effect of Medicare or private insurance was strongest in low poverty neighborhoods, less strong in moderately poor neighborhoods and nonexistent in high poverty neighborhoods. This same pattern of mediated and moderated effects was also observed for stage at diagnosis, waits for adjuvant radiation therapy and for receipt of sentinel lymph node biopsies. These findings are consistent with the theory that more facilitative social and economic capital is available in more affluent neighborhoods where women with breast cancer may be better able to absorb the indirect and direct, but uncovered, costs of care.

We did not find support for our hypothesis that Medicaid would also mediate the effect of poverty as we had for women with colon cancer [27]. However, this study’s sample of women with breast cancer and the previous study’s sample of women with colon cancer were demographically distinct. The women with breast cancer were much younger. They were twice as likely to be of pre-retirement age and 35% more likely to be covered by a commercial insurer. Younger women with breast cancer, more than half of whom had private health insurance, benefited greatly from having it. Whereas, older women with colon cancer, two-thirds of whom did not have private insurance, seemed to benefit relatively more from Medicaid coverage.

Medicare or private insurers were the primary payers for the care of eight out of every ten women in this study. It seems that the effectiveness of these, most prevalent, health insurance programs, public and private, are significantly impacted by the availability of other resources. In more well to do neighborhoods where social and economic capital abounds most women with breast cancer seem able to absorb the uncovered costs of cancer care. But high poverty neighborhoods, with their relative lack of such capital reserves, seem to remain places of “true disadvantage” [6], especially for the women who live there. Not only are they much more likely to be uninsured or underinsured, but even when insured such insurance programs seem to be less effective for them there than they are in less impoverished neighborhoods.

As for treatments, main and moderator effects indicative of protection, particularly in high poverty neighborhoods were observed for women with private health insurance. All of the following benefits of having commercial coverage among women living in high poverty neighborhoods were observed: shorter surgical waits, better access to BCS as well as to adjuvant radiation, chemo- and hormone therapies. And for women who received mastectomies, those with private insurance were more than twice as likely to have also received BRS.

These findings may seem counter hypothetical, but an extension of our health insurance-social capital theory could explain them. In such high poverty neighborhoods, having private health insurance could itself be deemed an important form of social capital. Especially in such vulnerable places, private health insurance seems to operate to the advantage of its holders. One way that it may operate is through its probable association with higher quality primary care. Privately insured women are more likely to have more established, continuous relationships with primary care physicians. Such relationships are known to provide myriad benefits: preventive surveillance, referral to and follow-up of specialist care, and advocacy and coordination throughout care processes [60,61]. Clearly, such could make a very big difference in the life of a woman with breast cancer, especially if she otherwise has limited personal and community resources available to her. This is not to say that high poverty neighborhoods are devoid of such resources. For example, evidence has been accumulating about the possible health benefits of living in certain relatively homogeneous communities such as Mexican American barrios [62,63]. Though such ethnic concentrations tend to be associated with concentrations of poverty, it seems that people and institutions in these communities provide quite a bit of effective social and economic support to one another. Despite their resiliencies though, gaps in access to key resources including primary care have been identified there. So it stands to reason that private insurance and attendant primary care could operate to potentiate the strengths and resiliencies that already exist in such high poverty neighborhoods.

### Payer effect sizes

This study’s payer effect sizes estimated with standardized rate ratios ranged from 1.06 to 2.25. Those most hypothetically central, the effects of having Medicare or private insurance on early diagnosis and survival among women with node negative breast cancer in high poverty neighborhoods, ranged from 1.10 to 1.19. Though significant in a statistical sense, they might be thought small for clinical practice or policy guidance. We think such would be an interpretive error for the following reasons. The attribution of risk at the population level is a function of three factors of which effect size is only one. It is also important to consider the size of the population at risk as well as the prevalence of exposures to the risk factors being studied. In this instance, the central exposure or risk factor to be mediated is a social one, that is, residence in a high poverty neighborhood. The other social exposure of interest or risk factor is the risk of being inadequately insured, that is, of being insured by Medicaid or uninsured. More than forty percent of the non-elderly, non-disabled California population or more than 13 million people are so affected. The national estimate approaches 100 million [64-66]. At the time of this study approximately 34 million Americans were poor and nearly 10 million of them lived in high poverty neighborhoods [35,67]. Regrettably, being uninsured, Medicaid insured or poor are not rare “exposures” in California or America. And nearly a quarter of a million American women are newly diagnosed with breast cancer each year [68]. So a change in relative risk of ten to twenty percent could affect more than 10 thousand women with breast cancer in California and nearly 50 thousand women in the United States each year. In terms of population attributable risk, we would deem these extraordinarily large effects.

### Potential limitations

Our use of ecological poverty measures might suggest alternative explanations for our results. One may wonder if the racial/ethnic composition of high poverty neighborhoods, rather than their concentration of the poor, accounted for the care and survival differences we observed. We do not think so for the following reasons. First, numerous recent US studies of breast cancer care and survival have found that socioeconomic differences explain most racial-group differences [69-74]. Second, after we accounted for age, and stage as well as the main and interacting effects of poverty and health insurance in these analyses, race/ethnicity was not significantly associated with survival. And third, our research group observed that health insurance disparities can explain essentially all of the breast cancer care disparities observed among our sample’s largest ethnic minority group (15.7%), Mexican Americans [75,76]. In short, while not refuting the importance of race, ethnicity and culture in cancer care [77-79], our analyses suggest that having adequate health insurance is probably much more important.

There has been a large unmet need for research on the validity of ecological measures of SES often used in public health research. Wilson and Jargowsky and others have added much knowledge on high poverty neighborhood measures [6-8,35,36] and our research group has done work to advance understandings of poor neighborhoods, analogous to this study’s middle poverty neighborhoods [80,81]. They seem prevalently represented by not only the poor, but the near poor and working poor as well as lower-middle and middle class people. This study added further to our knowledge about vulnerable neighborhoods. For example, steep gradients were observed for various types of health insurance across low (e.g., baseline Medicaid coverage) to high poverty neighborhoods (prevalence more than 12-fold greater [82,83]). This new knowledge about the validity of ecological poverty measures may advance our understandings about the contexts in which very poor Americans live. Therefore, our findings are probably not prone to ecological fallacy. They may, in fact, help researchers to better understand contextual measures of SES and so avoid individualistic fallacies of inference [84,85].

Another possible limitation of our study was incomplete information on outpatient treatments [51,86]. Such data are more difficult for cancer registries to collect than inpatient data. However, the California breast cancer registry has been enhanced well beyond the norm and has been shown to be nearly complete for chemotherapy data [34,87]. In addition, analyses of disease stage, hospital-based surgery and survival were unlikely to have been affected [88], and missing radiation therapy and chemotherapy data were not prevalent in our sample. Finally, we focused on overall survival, rather than cancer-specific survival. Although vital status and length of overall survival are highly accurate in cancer registries, the underlying cause of death probably is not [89-91]. Although death certificate error was a likely limitation, we did systematically replicate our central all-cause survival hypothesis tests with cancer-specific ones. If anything, our overall survival effects were slight underestimates of cancer-specific ones [90]. And the underlying cause of many deaths not coded as cancer mortality can be directly associated with lack of treatment or with cancer treatment complications [92]. For all of these reasons, we think overall survival a valid and policy-telling outcome. And such would clearly have had no impact on other indices of breast cancer care such as diagnoses and treatments.

## Conclusions

America’s multi-tiered health insurance system mediates the quality of breast cancer care. The system is inequitable and unjust as it advantages the well insured and the well to do. Recent health care reforms ought to be enacted across all 50 states in ways that are consistent with their federal legislative intent, that high quality health care be truly available to all.

## Human participants ethical review

This study was reviewed and cleared by the University of Windsor’s research ethics board.

## Competing interests

The authors declare that they have no competing interests.

## Authors’ contributions

KMG conceptualized and supervised the study and led the writing. KMG, INL, EJH, GYZ and CH obtained funding. GYZ supervised the analysis. All authors assisted with study design, data analysis and interpretation, and writing. All authors read and approved the final manuscript.
